# Copy number variation of urine exfoliated cells by low-coverage whole genome sequencing for diagnosis of prostate adenocarcinoma: a prospective cohort study

**DOI:** 10.1186/s12920-022-01253-5

**Published:** 2022-05-05

**Authors:** Youyan Guan, Xiaobing Wang, Kaopeng Guan, Dong Wang, Xingang Bi, Zhendong Xiao, Zejun Xiao, Xingli Shan, Linjun Hu, Jianhui Ma, Changling Li, Yong Zhang, Jianzhong Shou, Baiyun Wang, Ziliang Qian, Nianzeng Xing

**Affiliations:** 1 Department of Urology, National Cancer Center/National Clinical Research Center for Cancer/Cancer Hospital, Chinese Academy of Medical Sciences and Peking Union Medical College, Beijing, 100021 China; 2 State Key Laboratory of Molecular Oncology, National Cancer Center/National Clinical Research Center for Cancer/Cancer Hospital, Chinese Academy of Medical Sciences and Peking Union Medical College, Beijing, 100021 China; 3 Cancer Hospital of Huanxing, ChaoYang District, Beijing, 100122 China; 4 Hongyuan Biotech, Biobay, Suzhou, China

## Abstract

**Background:**

Non-invasive, especially the urine-based diagnosis of prostate cancer (PCa) remains challenging. Although prostate cancer antigen (PSA) is widely used in prostate cancer screening, the false positives may result in unnecessary invasive procedures. PSA elevated patients are triaged to further evaluation of free/total PSA ratio (f/t PSA), to find out potential clinically significant PCa before undergoing invasive procedures. Genomic instability, especially chromosomal copy number variations (CNVs) were proved much more tumor specific. Here we performed a prospective study to evaluate the diagnostic value of CNV via urine-exfoliated cell DNA analysis in PCa.

**Methods:**

We enrolled 28 PSA elevated patients (≥ 4 ng/ml), including 16 PCa, 9 benign prostate hypertrophy (BPH) and 3 prostatic intraepithelial neoplasia (PIN). Fresh initial portion urine was collected after hospital admission. Urine exfoliated cell DNA was analyzed by low coverage Whole Genome Sequencing, followed by CNV genotyping by the prostate cancer chromosomal aneuploidy detector (ProCAD). CNVs were quantified in absolute z-score (|Z|). Serum free/total PSA ratio (f/t PSA) was reported altogether.

**Results:**

In patients with PCa, the most frequent CNV events were chr3q gain (n = 2), chr8q gain (n = 2), chr2q loss (n = 4), and chr18q loss (n = 3). CNVs were found in 81.2% (95% Confidence Interval (CI) 53.7–95.0%) PCa. No CNV was identified in BPH patients. A diagnosis model was established by incorporating all CNVs. At the optimal cutoff of |Z|≥ 2.50, the model reached an AUC of 0.91 (95% CI 0.83–0.99), a sensitivity of 81.2% and a specificity of 100%. The CNV approach significantly outperformed f/t PSA (AUC = 0.62, *P* = 0.012). Further analyses showed that the CNV positive rate was significantly correlated with tumor grade. CNVs were found in 90.9% (95% CI 57.1–99.5%) high grade tumors and 60.0% (95% CI 17.0–92.7%) low grade tumors. No statistical significance was found for patient age, BMI, disease history and family history.

**Conclusions:**

Urine exfoliated cells harbor enriched CNV features in PCa patients. Urine detection of CNV might be a biomarker for PCa diagnosis, especially in terms of the clinically significant high-grade tumors.

**Supplementary Information:**

The online version contains supplementary material available at 10.1186/s12920-022-01253-5.

## Introduction

The mortality trend of prostate cancer (PCa) ranges widely from country to country in the world [1]. In recent years, PCa mortality has decreased in most western nations but increased in other countries such as China [2]. Currently, screening for PCa still remains one of the most controversial topics in the urological literature. But it is a common sense that we should try to find out clinically significant PCa [3, 4].

As we all known, prostate biopsy is the gold standard to diagnose PCa. Currently, PSA is the only PCa biomarker applied clinically. The need for prostate biopsy is mainly based on the prostate specific antigen (PSA) level and/or suspicious digital rectal exam (DRE) and/or imaging. Unfortunately, PSA screening tends to report frequent false positives [5]. To refrain from abusing the subsequent invasive procedures, factors including age, potential comorbidity, and therapeutic consequences should also be considered and discussed beforehand. The benefit of PSA screening could be outweighed by loss of quality-of-life owing to post-diagnosis long-term effects, as proved by a 11-year follow-up [6].

PSA is more of a tissue-specific marker than a cancer-specific marker; therefore, it may be elevated in benign prostatic hypertrophy (BPH), prostatitis, and other non-malignant conditions. Most men with elevated PSA levels do not have prostate cancer [7, 8]. Total PSA level > 10 ng/ml confers a greater than 67% likelihood of biopsy-detected prostate cancer, while only about 18% of men with 4–10 ng/ml PSA result in a positive biopsy [9]. In 2017 the US Preventive Services Task Force (USPSTF) issued an updated statement, suggesting that men aged 55–69 be informed about the benefits and harms of PSA-based screening, as this might be associated with only a small survival benefit [10].

PSA has a high sensitivity but a low specificity. To overcome the limitations, the free/total (f/t PSA) ratio appears as a more clinically useful biomarker to reduce the number of negative biopsies. However, the usefulness of f/t PSA is still controversial. Some studies reported that the f/t PSA ratio showed high diagnostic sensitivity (94%) and specificity (93%) [11]. Other studies, however, have reported much lower sensitivity of 75% and specificity of 32%. A systematic meta-analysis has been provided in recent publication [12].

We need a more ideal, non-invasive method to screen and diagnose PCa, especially clinically significant PCa [3]. The past decade witnessed exponential progress in non-invasive liquid biopsy for malignancies, e.g. the urine-based testing for prostate cancer [13]. PCa is notoriously known as a heterogeneous cancer entity with complex cell origins and mutation profiling, in which context urine sampling is especially attractive in capturing the tumor heterogeneity originating from multiple clonal populations. Through a systemic and aggregate perspective into genomic aberration, chromosomal instability (CIN) arises as a promising alternative diagnostic tool for PCa. CIN refers to ongoing aberrant chromosome segregation during cell division, generally exhibiting somatic copy number variation (CNV). As a hallmark of cancer, CIN is pervasive in almost 90% of solid malignancies. CIN also underpins much of the tumor heterogeneity and is central to cancer evolution [14, 15]. The effect CNV exerts on the cancer genome is suggested to far outweigh certain genetic mutations [16]. In our previous works, we applied CIN detection on urothelial cancer [17], liver cancer [18], breast cancer [19], bile duct carcinoma [20], etc. In particular, a previous study based on tumor tissues detected significant focal amplifications and deletions in PCa [21] It has been found that PCa patients with CIN are associated with lethal progression and worse prognosis [22, 23], which suggests the relevance of CIN with clinically significant PCa [3].

In this study, we employed low-coverage Whole Genome Sequencing (WGS) to study CNV in urine-exfoliated cell DNA, successfully demonstrating an alternative approach to non-invasive, highly sensitive and specific PCa screening.

## Methods

Our prospective study adhered to the Standards for Reporting Diagnostic Accuracy Studies (STARD) 2015 guideline. We continuously enrolled PSA-elevated patients who were suspected to have PCa and thus underwent transperineal prostate biopsy from Aug 2020 to January 2021 at Cancer Hospital, Chinese Academy of Medical Sciences.

### Inclusion and exclusion criteria

All the patients were outpatients who were referred to our hospital due to the abnormal PSA. PSA abnormality was defined by either of two criteira: (1) the total PSA was equal or above 10 mg/ml, or (2) the total PSA was the 4–10 mg/ml range and the f/t PSA was below 0.16. All the patients were informed of the potential harms and benefits of the biopsy due to the abnormal PSA. A sample of 50 ml fresh urine was collected before biopsy.

### DNA extraction

Total genomic DNA was isolated from urine exfoliated cell using the Amp Genomic DNA Kit (TIANGEN). Next-generation sequencing was performed as previously described [17]. DNA was fragmented into an average size of 190 bp, and then 100 ng of fragmented genomic DNA was used for preparation of sequencing libraries (NEBnext Ultra II). Barcoded sequencing adaptors of 8-bp length were ligated with DNA fragments and amplified by PCR. Purified sequencing libraries were massively parallel sequenced by Illumina HiSeq X Ten platform. Raw sequencing data amounted to about 5G per sample, and they were quality controlled and aligned to the human reference genome version HG19.

### Low-coverage WGS and prostate cancer chromosomal aneuploidy detector (ProCAD)

The current study practiced low-coverage WGS to maximize the efficiency-to-cost ratio. For low-coverage WGS, libraries were prepared using the Kapa Hyper Prep kit with custom adapters (IDT and Broad Institute) starting with 3–20 ng of DNA input (median, 4 ng), or approximately 800–5000 haploid genome equivalents. Up to 16 libraries were pooled and sequenced using 150 bp paired-end runs over 1× lane on a HiSeq ×10 (Illumina). Chromosomal CNVs were identified via the customized workflow Prostate Cancer Chromosomal Aneuploidy Detector (ProCAD). Poor-quality sequence data would be flagged if the median absolute deviation of copy ratios (log2 ratio) between adjacent bins, genome-wide, was 0.38, and the corresponding sample would be excluded in such a case. ProCAD test results were blinded against the clinical professionals.

Urine exfoliated cell DNA was extracted and analyzed by Illumina ×10. At least 15,000,000 paired reads were collected for each sample. The reads were mapped to human reference genome HG19. Genomic coverage was counted using the software SAMtools mpileup [24] Then we calculated average coverage for each 200-kb bin. Z-scores for each bin was then normalized by Z-score by using Formula:

$$Z = \frac{{V_{tumor} - average\left( {V_{control} } \right)}}{{stdev\left( {V_{control} } \right)}}$$

The Circular binary segmentation (CBS) algorithm accessible from R package DNACopy [25] was used to find significant genomic breakpoints and copy number changed genomics segments.

Categorical variables were reported as frequencies and percentages, and continuous variables were described as mean and standard deviation (SD), or median with interquartile range (IQR), as appropriate. Continuous variables were analyzed using the Mann–Whitney U test, and categorical variables were analyzed with Fisher’s exact test, as appropriate. Missing data were discarded from analyses. All analyses were performed with SPSS18.0 (SPSS Inc., Chicago, IL, USA), R software (version 3.4.3; R Foundation for Statistical Computing), and MedCalc software (version 19.1; MedCalc Software, Mariakerke, Belgium). *P* < 0.05 was considered as statistically significant.

## Results

### Patient characteristics

**Fig. 1:**
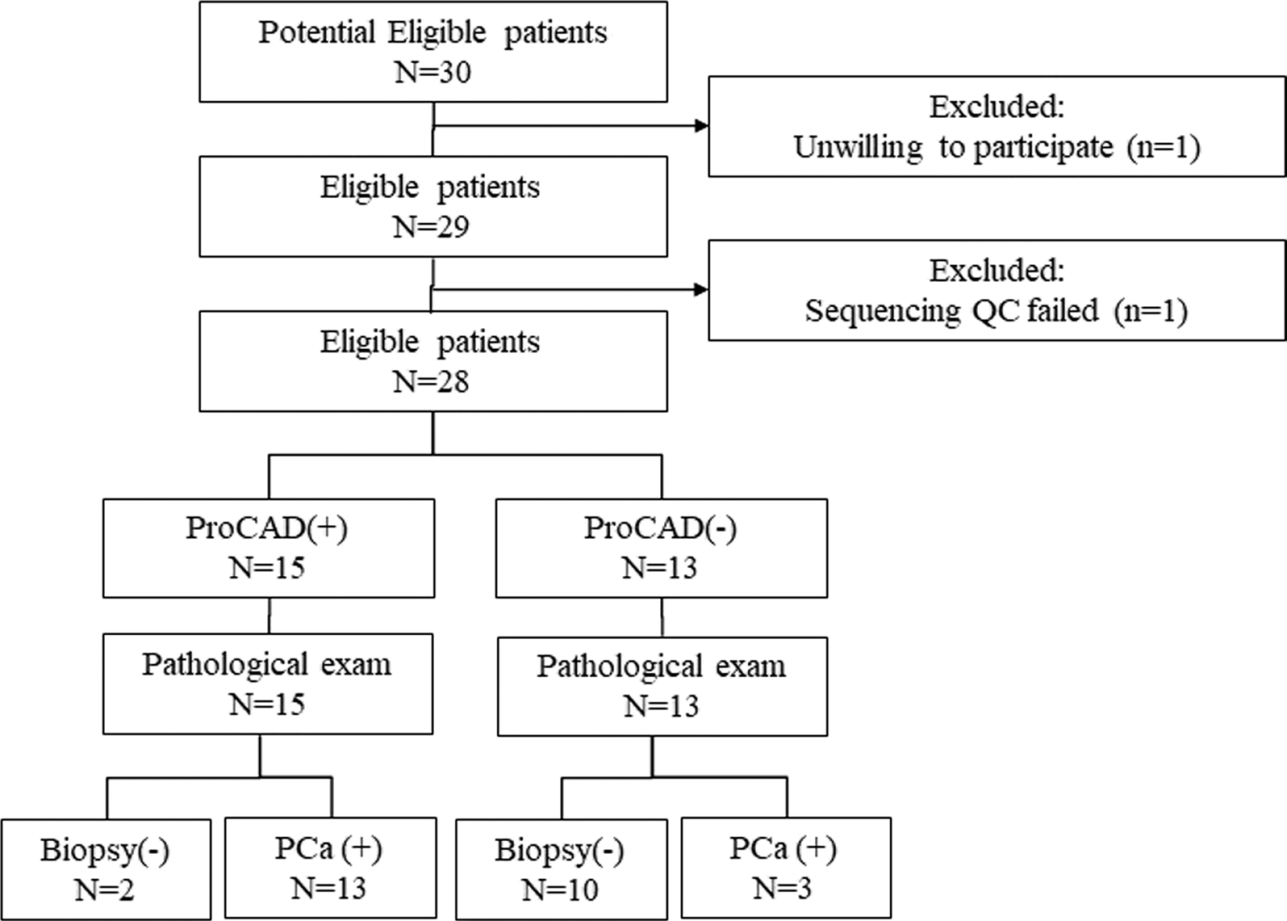
The STARD flowchart for participants recruitment

Between Mar 2019 and Jan 2020, 30 consecutive patients who were suspected of PCa with moderate to high confidence were admitted to our department. The STARD flow diagram was shown in Fig. 1. Among them, one patient declined biopsy, and one urine sample failed sequencing quality control. Finally, a total of 28 patients with prostate conditions were eligible for analysis. The median age was 68 years (Table 1). PSA level, free/total PSA ratio (f/t PSA) and DRE finding were shown for each patient also listed in Table 1. On pathological examinations, 16 (57.1%) patients had prostate adenocarcinoma, with Gleason score 3 + 3, 3 + 4, above 4 + 3 in 4 patients, 1 patient and 11 patients respectively. Twelve patients had post-biopsy benign findings: BPH in 9 patients and Prostatic Intraepithelial Neoplasia (PIN) in 3 patients. In terms of baseline characteristics, such as age, BMI, smoking history, and family history, there was no statistical difference between benign patients and cancer patients (Additional file 1).

**Table 1:** Baseline characteristics of the included patients

ID	Age	PSA			16-needle biopsy pathology
		PSA1	PSA2	f/t PSA	
0009315	68	11.30	4.97	21.63	BPH
0009901	60	9.81	10.67	19.82	BPH
0009750	74	14.17	10.99	19.09	BPH
SH-CL	57	7.96	7.76	8.88	BPH
0009973	70	23.32	15.20	15.75	BPH
0010268	65	14.38	9.18	9.52	BPH
0009994	56	11.39	8.35	14.65	BPH
0010622	65	10.24	9.26	13.61	BPH
SH-ZRM	62	7.70	6.81	23.66	BPH
0010253	56	13.11	13.88	21.13	PIN
0010244	69	7.29	6.70	8.74	PIN
0010247	60	7.11	5.64	22.06	PIN
0009552	69	> 1000	1000.00	9.60	PCa, GS = 3 + 3 = 6
0009330	79	121.00	151.00	12.45	PCa, GS = 3 + 3 = 6
SH-WSG	70	7.98	8.68	11.11	PCa, GS = 3 + 3 = 6
SH-ZHJ	73	8.41	3.85	18.72	PCa, GS = 3 + 3 = 6
0009245	73	80.57	76.09	22.67	PCa, GS = 3 + 4 = 7
0010245	65	15.13	14.60	8.15	PCa, GS = 4 + 3 = 7
SH-LHY	74	7.82	6.70	26.27	PCa, GS = 4 + 3 = 7
0009210	63	486.00	343.80	–	PCa, GS = 3 + 5 = 8
0010753	77	14.10	12.64	23.82	PCa, GS = 4 + 4 = 8
0009675	74	13.90	11.59	5.65	PCa, GS = 4 + 4 = 8
SH-JBL	64	424.60	555.01	–	PCa, GS = 4 + 4 = 8
SH-ZYX	73	33.07	29.76	21.57	PCa, GS = 4 + 5 = 9
0009884	70	260.50	236.28	–	PCa, GS = 4 + 5 = 9
0009834	69	> 1000	56.83	–	PCa, GS = 4 + 5 = 9
0009551	73	819.00	602.99	–	PCa, GS = 4 + 5 = 9
0009476	79	> 1000	1000.00	–	PCa, GS = 5 + 4 = 9

### CNV profile

**Fig. 2:**
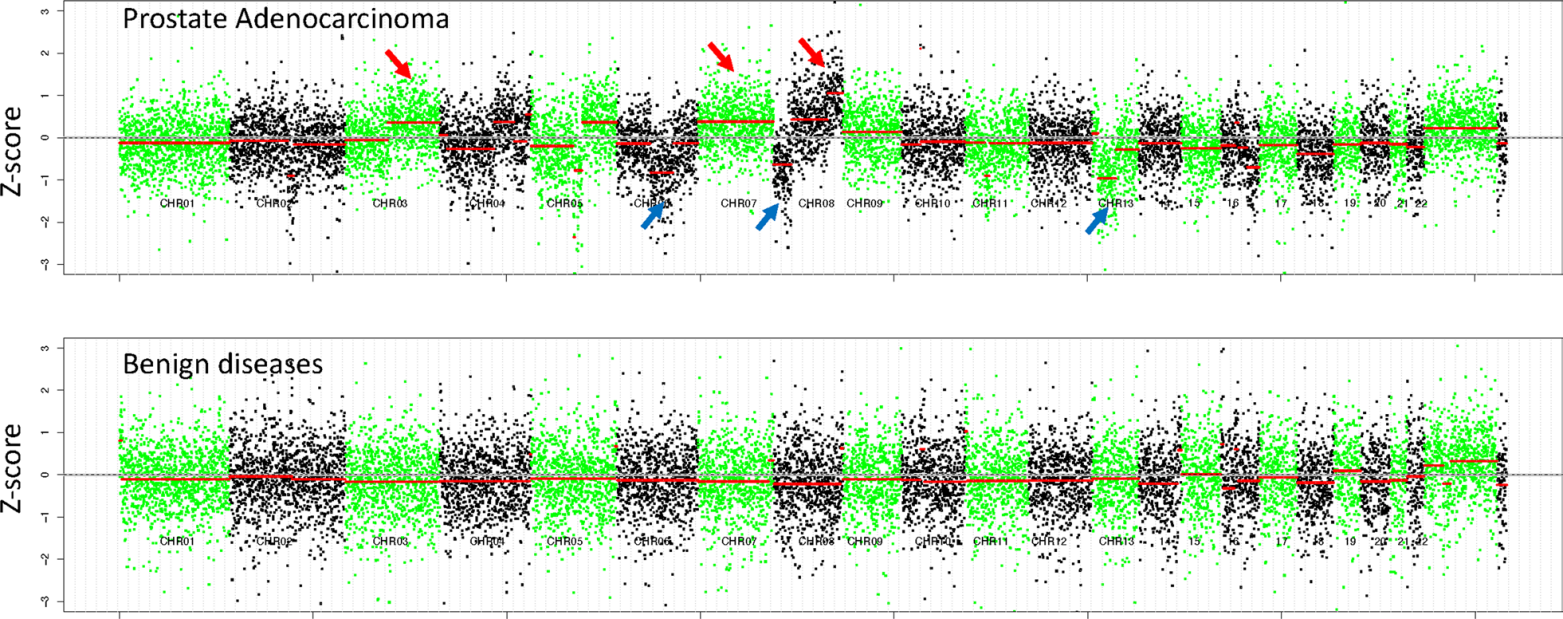
Overview of copy number variations via urine exfoliated cell DNA analysis patients. **a** genome overview of prostate cancer **b** genome overview of benign samples. Red arrows indicate marked CNV gains in the cancer genome

All urine exfoliated cells samples passed sequencing data quality assessment. The positive rate of CNV was in 81.3% (13/16) in patients with PCa and 0.0% (0/9) in patients with benign prostate hypertrophy. The genome-wide landscapes of CNVs in all patients were shown in Fig. 2, A and B. Representative chromosome CNVs included 2q loss (n = 4), 18q loss (n = 3), 13q loss (n = 1), 16q loss (n = 1), 7q gain (n = 4), 3q gain (n = 2) and 8q gain (n = 2) in prostate adenocarcinoma (Fig. 2a). None of these CNVs were found in BPH (Fig. 2b). Z-scores for all 44 arms of autosomal chromosomes were calculated with normalization to benign controls. A heatmap of Z-scores was generated (Additional file 2). Cancer patients had more genome aberrations than BPH patients. More chromosomal gains were found in high grade tumors (Gleason score ≥ 4 + 3, Additional file 2). Four (36.4%) high grade tumors were found with more than one chromosomal CNV (|Z|≥ 2.5). Meanwhile, only one (20.0%) low grade tumor harbored more than one CNV (Additional file 2).

### Z-scores between PCa and BPH

**Fig. 3:**
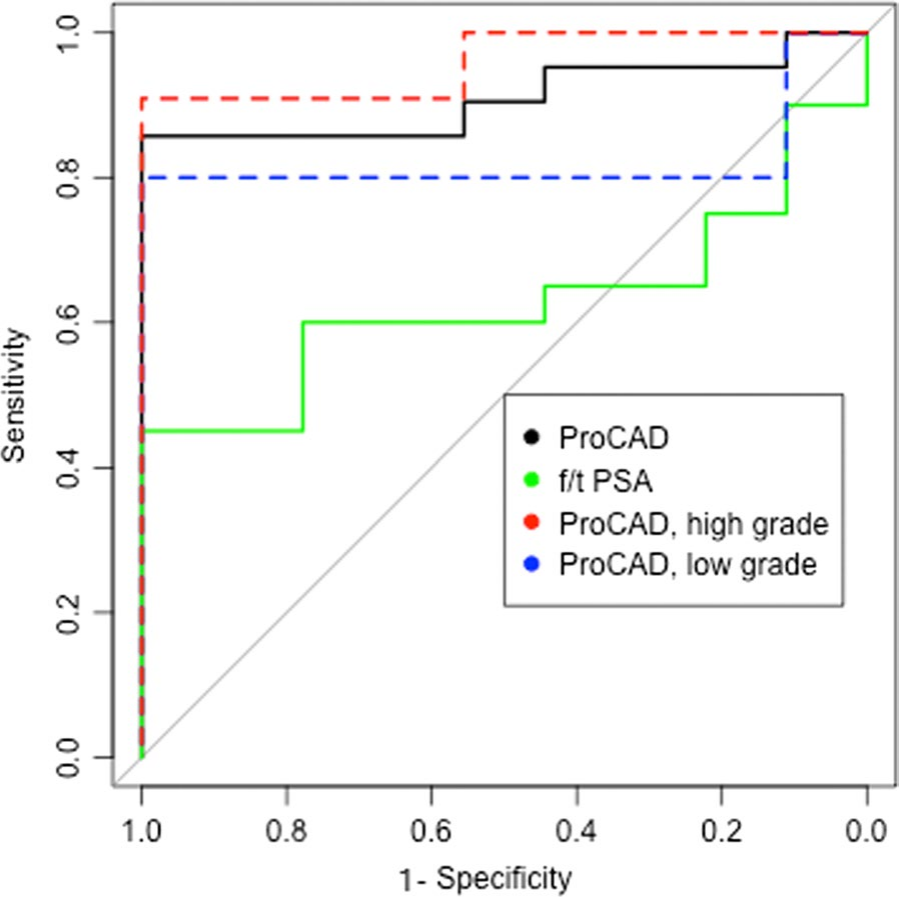
Diagnostic performance of chromosome Z-scores in the form of receiver–operating-characteristic curves

We further explored the value of Z-score of each chromosome arm in differentiating PCa from BPH. The AUCs ranged from 0.44 to 0.785 (median AUC = 0.62, Table 2). Chr8q showed high diagnostic accuracy, with an AUC of 0.76 (Table 2). We combined information of all chromosomes to build a diagnostic model for PCa. The optimal Z-score cutoff |Z|≥ 2.50 was calculated by Youden Index. At this cutoff, ProCAD test showed a sensitivity of 81.3% and a specificity of 100% (Table 3). The AUC was 0.91 (95% CI 0.83–0.99) (Fig. 3), which was better than the result from any single chromosome. A lower cutoff (|Z|≥ 2) showed better sensitivity (94.1%), while compromising the specificity (37.5%). Compared with traditional tumor biomarker of serum f/t PSA, the overall diagnostic accuracy of ProCAD test demonstrated a significantly higher performance, with AUC 0.91 versus 0.62 (*P* = 0.012, Table 3).

**Table 2:** Diagnostic performance of each chromosomal arms

Marker	AUC	95% CI
chr12q	0.775	[0.54, 1.00]
chr2q	0.769	[0.54, 0.99]
chr8q	0.763	[0.58, 0.94]
chr7q	0.744	[0.53, 0.94]
chr3q	0.700	[0.47, 0.92]
chr8p	0.700	[0.48, 0.91]
chr19q	0.697	[0.49, 0.89]
chr15q	0.694	[0.44, 0.94]
chr19p	0.694	[0.45, 0.93]
chr21p	0.694	[0.45, 0.93]
chr1q	0.688	[0.42, 0.95]
chr3p	0.669	[0.44, 0.89]
chr21q	0.663	[0.41, 0.90]
chr16p	0.656	[0.41, 0.89]
chr10p	0.644	[0.42, 0.86]
chr20p	0.638	[0.39, 0.87]
chr9q	0.631	[0.42, 0.83]
chr4p	0.625	[0.39, 0.85]
chr2p	0.619	[0.39, 0.84]
chr18q	0.613	[0.38, 0.83]
chr20q	0.613	[0.37, 0.84]
chr13q	0.606	[0.38, 0.82]
chr11q	0.600	[0.37, 0.82]
chr12p	0.600	[0.36, 0.83]
chr11p	0.588	[0.33, 0.84]
chr4q	0.581	[0.34, 0.81]
chr5q	0.569	[0.35, 0.78]
chr16q	0.563	[0.33, 0.79]
chr7p	0.550	[0.30, 0.79]
chr17p	0.550	[0.29, 0.80]
chr5p	0.538	[0.28, 0.79]
chr10q	0.538	[0.29, 0.78]
chr1p	0.531	[0.28, 0.77]
chr14q	0.519	[0.27, 0.76]
chr6q	0.500	[0.28, 0.71]
chr9p	0.488	[0.21, 0.76]
chr17q	0.456	[0.22, 0.69]
chr18p	0.450	[0.20, 0.69]
chr6p	0.438	[0.18, 0.69]

**Table 3:** Diagnostic performance of ProCAD by incorporating all chromosomes

	AUC (95% CI)	Cutoff	TN	TP	FN	FP	PPV (%)	NPV (%)	Specificity (%)	Sensitivity (%)	Accuracy (%)
ProCAD CHR1-22	0.91 (0.83, 0.99)	2	3	15	1	6	71.4	75.0	33.3	93.8	72.0
		2.5	9	13	3	0	100.0	75.0	100.0	81.3	88.0
		3	9	8	8	0	100.0	52.9	100.0	50.0	68.0
f/t PSA (%)	0.63 (0.42, 0.83)	< 10%	7	9	7	2	81.8	50.0	77.8	56.3	64.0
		< 16%	4	11	5	5	68.8	44.4	44.4	68.8	60.0

ProCAD versus f/t PSA, *P* = 0.012

Correlation between ProCAD test positivity and patient clinicopathological features was explored. Gleason score tends to correlate with ProCAD urine test positivity (*P* value = 0.038). The diagnosis model identified 90.9% (11/12) of the high-grade cancers, and 60.0% (3/5) of the low-grade tumors. There was history of hormone therapy for the single high grade tumor missed by ProCAD. The sensitivity for high grade tumor would be 100% if this case had been excluded. Other parameters, including TNM stages, vascular invasion, lymph node metastasis, or distant metastasis were not significantly associated with ProCAD test positivity (data not shown).

**Fig. 4:**
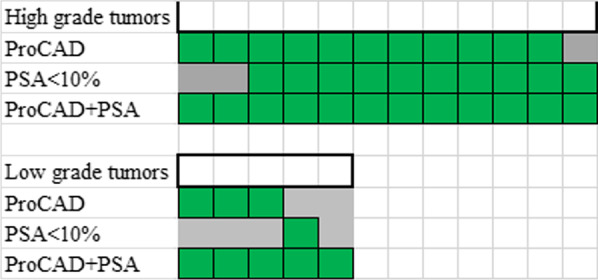
Diagnostic performance by combing both PSA and ProCAD. Each column represents one high-grade prostate tumor. Green shade indicates correct diagnosis

As shown in Fig. 4, we also examined the diagnostic value of adding f/t PSA to the ProCAD test. The sensitivity for the combined test was 100% (11/11) and 80.0 (4/5) for high-grade and low-grade tumors, respectively, which tended to be better than ProCAD test alone (*P* = 0.71).

## Discussion

By analyzing 16 patients with prostate cancer (PCa) and 9 patients with benign prostatic hypertrophy (BPH), this pilot study showed that the sensitivity and specificity of ProCAD test in diagnosing PCa reached 81.3% and 100%, respectively. Overall, this test was significantly superior to f/t PSA (AUC 0.91 vs. 0.62, *P* = 0.012). This study also represents a major step forward from previous work, which generally detected limited number of cancer-related genetic mutations or only used panel-based gene mutation of methylation tests [26]. By contrast, CNV plays as a systemic summary of abundant and mixed genetic aberrations that reflect genome instability, tumor heterogeneity and clone evolution [15]. This was the first study to systematically describe arm-level CNVs across the whole genome via urine exfoliated cell DNA in PCa patients. The value of CNV profile as diagnostic tool was also explored.

In patients with suspicious findings of the prostate, PSA is always firstly tested to guide diagnosis. Nevertheless, the diagnostic value of PSA is far from accurate, even though it is regarded as a textbook serum marker for PCa. It was suggested as more of a tissue-specific biomarker than a cancer-specific biomarker [27]. Chronic conditions, such as inflammation and other stimulations may also promote the damage of prostate gland cells and thus increase blood level of PSA. Hence, PSA testing has caused exceedingly biopsies, intensified anxieties, and reduced quality of life [6, 28]. Some investigators attempted to improve the diagnostic accuracy by increasing the cutoff of total PSA. In our results by using a cut-off of 50 ng/ml, the specificity increased to 100%, yet at a cost of substantially decreased sensitivity of 50.0% (data not shown). Falsely increased PSA levels have indeed misled us to perform biopsies, which eventually reveal benign diseases. People also considered f/t PSA to increase the diagnosis accuracy [12, 29]. In our research, the specificity was increased to 77.8% by using f/t PSA cutoff ≤ 10%, yet at a cost of substantially decreased sensitivity 56.3% (Table 3). Even so, the specificity was still not good enough to limit unnecessary biopsies. As reported in previous researches, chromosomal instability (CIN) is a much more tumor-specific biomarker [30, 31]. In the current study, the ProCAD test showed much better performance as compared to f/t PSA, with specificity of 100% and sensitivity of 81.3% (*P* = 0.012). Our study suggests that, rather than f/t PSA, a urine based CNV analysis in addition to serum PSA may help clinicians identify PCa more specifically.

It is a routine practice to perform biopsy evaluation for patients with elevated PSA, which always accompanies histopathological examinations in order to increase the diagnostic yield. This practice tends to diagnose an increasing proportion of low-grade tumors. Patients with low grade tumors usually showed good survival [32]. The application of ProCAD technique in PCa is endorsed by the fact that this malignancy is particularly rich in CNVs. Human cancers can be divided into two groups based on oncogenic signatures: M class (primarily with mutations) and C class (primarily with copy number alterations). TP53 mutation was regarded as a typical feature of C-class cancers [33]. In patients with PCa, TP53 mutation is frequently seen, and is more predominant in high grade tumors cancer [34]. With regards to our cohort, the whole-genome study revealed that high grade tumors exhibit particularly high copy-number alterations. In this research, the urine-based diagnosis methods showed much higher sensitivity for high grade tumors as compared to low grade ones (91% vs. 60%, *P* = 0.0055). The data show that patients with elevated serum PSA and a positive urine CNV finding would potentially be at risk for more aggressive prostate cancers, which requires taking action immediately. For patients with negative urine findings, it might be a low-grade tumor and it warrants another PSA testing to further confirm the aggressiveness before taking action.

Our study had several advantages. This study was prospectively designed, and the conduct and report of tests were done blinded against the clinical professionals. Thus, we avoided the problem of hypothesis-generating data in most retrospective or non-blinded studies. Compared with panel-based high-depth sequencing, low-coverage WGS of urine cell DNA markedly decreased the cost and shortened testing duration. By covering the whole genome, this method detected larger number of chromosomal rearrangements. Furthermore, our cohort included a group of patients with heterogeneous prostate etiologies and thus indicated the generalizability of ProCAD test in the real-world practice. Since many patients tend to avoid invasive procedures and would prefer a urine test, our test could fill a critical niche for strengthening surveillance among high-risk individuals with benign lesions and/or pre-cancer lesions. As far as we know, this was the first time that a non-invasive and comprehensive chromosomal analytic approach was introduced into PCa diagnosis.

A perfect bio-marker is like the distant holy grail in tumor diagnosis. Several reasons may add noise to the diagnostic accuracy of ProCAD. Firstly, the sensitivity of low-coverage WGS is not as high as deep-sequencing and it is more useful with a relatively high tumor fraction. The fraction of tumor cells from prostate is low in the urine. Secondly, given the adverse impact of COVID-19 for patient recruitment, the enrollment process was slow and thus the sample size was not sufficiently large. There may be a chance for a type I error. The small number of cases also precluded us from verifying distinct CNV patterns for different types of PCa. The favorable pilot results of this study may promote more urologist to participate in future multicenter validation.

Notably, few of our patients had history of PSA between 4 and 10 U/ul, which is the major focus of prostate cancer screening research space. Thus, the sample representativeness may be compromised. Furthermore, due to cost concern, we only provided urine cell results, but did not carry out confirmation by tissue analyses. As our study focused on diagnosing PCa at the chromosomal level, we did not further crack the genetic codes that delineated the formation of CNVs. This work can be advanced in future based on previous evidence with respect to the genomic landscape of PCa. In some PCa patients, the carcinogenesis may be primarily driven by specific genetic mutations, and ProCAD may incidentally miss positive findings in this subgroup.

## Conclusions

Our study demonstrates a proof of concept for the feasibility of ProCAD test discriminating benign prostate diseases from malignancies. The ProCAD test is a useful adjunct test to PCa in clinical practices. As a non-invasive, cost-effective, and time-saving method, it serves as a particularly useful complementary diagnostic tool for patients with long-term precancerous prostate conditions and outpatients under active surveillance program.

## Supplementary Information

**Additional file 1.** Detailed baseline information for patients recruited.

**Additional file 2.** List of copy number variations of each sample.

## Data Availability

The datasets as well as custom R code generated during the current study are available from the corresponding author on reasonable request. The raw sequence and processed data files are available through the National Omics Data Encyclopedia database (https://www.biosino.org/node/search) with accession number OEP002886.

## Abbreviations

BPH: Benign prostatic hypertrophy
CIN: Chromosomal instability
CNV: Copy number variation
DRE: Digital rectal exam
PCa: Prostate cancer
PIN: Prostatic intraepithelial neoplasia
ProCAD: Prostate cancer chromosomal aneuploidy detector
PSA: Prostate specific antigen
WGS: Whole-genome sequencing
